# Case Report: Successful conversion surgery following chemo-antiangiogenic therapy in a lung adenocarcinoma patient with active rheumatoid arthritis and rare EGFR mutation after immunotherapy-triggered flare

**DOI:** 10.3389/fonc.2026.1795914

**Published:** 2026-06-08

**Authors:** Junfeng Ma, Peng Yan, Chonghua Liu, Yuanyuan Wang

**Affiliations:** Department of Oncology, Linyi County People’s Hospital, Dezhou, China

**Keywords:** adverse events, bevacizumab, conversion surgery, immune checkpoint inhibitors, non-small cell lung cancer, rheumatoid arthritis, uncommon EGFR mutation

## Abstract

**Background:**

First-line treatment strategy for advanced non-small cell lung cancer (NSCLC) patients with concurrent driver mutations, high PD-L1 expression, and active autoimmune disease poses a significant challenge with limited guidance.

**Case presentation:**

A 41-year-old male with newly diagnosed active rheumatoid arthritis (RA) was found to have right lower lobe adenocarcinoma with ipsilateral interlobar pleural nodules (cT4N0M1a, stage IVA). Molecular profiling revealed a non-classical EGFR exon 18 mutation (E709_T710>del) with TP53 co-mutation and high PD-L1 expression (TPS 55%). Initial therapy with ‘pemetrexed + carboplatin + tislelizumab’ was administered. However, after only one cycle, the patient experienced a significant flare of RA symptoms and worsened serological markers, necessitating permanent discontinuation of immunotherapy. The RA flare was managed with methylprednisolone 1 mg/kg/day followed by a slow prednisone taper and sulfasalazine. Treatment was switched to ‘pemetrexed + carboplatin + bevacizumab’. After six cycles, restaging by contrast-enhanced CT showed partial response (PR) of the primary lesion and pleural metastases. Preoperative PET-CT demonstrated complete metabolic response of pleural nodules. Following multidisciplinary team (MDT) assessment and adherence to a strict 6-week preoperative bevacizumab washout period (based on pharmacokinetic reasoning and NCCN guidelines, though prospective data are lacking), the patient underwent conversion from thoracoscopic to open right lower lobectomy with systematic lymph node dissection due to severe fibrosis and frozen hilar lymph nodes. Postoperative pathology confirmed downstaging to ypT2N1 (1 of 12 positive lymph nodes), with a tumor regression grade (TRG) of 1 according to IASLC 2020 criteria (residual tumor cells <10%, significant regression). Postoperative maintenance therapy with ‘bevacizumab + pemetrexed’ was continued for 12 cycles, and the patient remains disease-free with a progression-free survival (PFS) of 20 months at last follow-up (on a regular imaging surveillance schedule).

**Conclusion:**

For advanced NSCLC patients with active autoimmune disease, immune checkpoint inhibitor therapy requires extreme caution even in the presence of high PD-L1 expression. Chemotherapy combined with antiangiogenic therapy can be an effective and safe induction strategy in this complex population. It may create a potential opportunity for conversion surgery in well-responding patients, enabling long-term disease control. This case underscores the critical importance of highly individualized, multidisciplinary treatment decision-making.

## 1 Introduction

The treatment for advanced non-small cell lung cancer (NSCLC) has entered an era of molecularly driven precision medicine. For patients with classical EGFR mutations (e.g., exon 19 deletions, L858R), EGFR tyrosine kinase inhibitors (TKIs) are the established standard first-line therapy. However, optimal first-line strategies remain controversial for non-classical or uncommon EGFR mutations (e.g., G719X, L861Q, S768I, and E709X), which constitute approximately 10-15% of all EGFR mutations. This complexity is amplified when these mutations co-exist with high PD-L1 expression (1). Furthermore, a significant proportion of lung cancer patients present with comorbid autoimmune diseases (AIDs), such as rheumatoid arthritis (RA). Although clinical trials often exclude patients with active AIDs, immune checkpoint inhibitors (ICIs) are increasingly used in real-world practice. ICIs are known to potentially induce or exacerbate AIDs, leading to treatment discontinuation and impacting oncological outcomes, making the initial treatment choice for NSCLC patients with active RA particularly challenging (2).

Currently, there is no standard treatment for the unique subgroup of patients with concurrent uncommon EGFR mutations, high PD-L1 expression, and active AID. Chemotherapy combined with antiangiogenic agents is a common clinical option, but its efficacy, safety, and potential as induction therapy for downstaging and subsequent conversion surgery in this population are rarely reported. This article details the case of a patient with active RA, an EGFR exon 18 (E709_T710>A) uncommon mutation, and high PD-L1-expressing advanced lung adenocarcinoma. After experiencing severe RA exacerbation triggered by initial chemo-immunotherapy, the patient achieved a significant response upon switching to chemotherapy combined with bevacizumab, subsequently underwent successful radical surgery, and attained long-term disease-free survival. This case aims to provide practical management experience and decision-making insights for this clinical dilemma.

## 2 Case presentation

### 2.1 History and diagnosis

A 41-year-old male, non-smoker, presented with swelling and pain in multiple limb joints in November 2023. Physical examination revealed limited mobility in the lower limbs without joint deformity. Laboratory tests showed an elevated erythrocyte sedimentation rate (ESR) of 87 mm/h, a high rheumatoid factor (RF) of 370 IU/ml, and a positive anti-cyclic citrullinated peptide (CCP) antibody of 34.5 RU/ml, leading to a diagnosis of RA. Concurrently, a chest CT scan, performed as part of related examinations for biological therapy, incidentally revealed a mass in the right lower lobe. Contrast-enhanced CT demonstrated an irregular right lower lobe mass (approx. 3.5cm × 2.8cm) with ill-defined borders to the diaphragm and heterogeneous enhancement, along with multiple small nodules along the right interlobar pleura (**Figure 1A**). Contrast-enhanced brain MRI was also negative for brain metastases. A CT-guided lung biopsy pathology confirmed moderately differentiated adenocarcinoma. Immunohistochemistry was positive for TTF-1 and NapsinA, with a Ki-67 proliferation index of approximately 60%.

**Figure 1:**
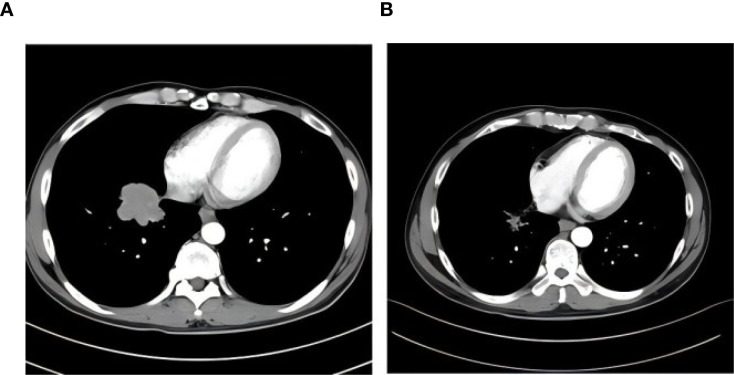
Imaging response to chemo-antiangiogenic therapy. **(A)** Baseline contrast-enhanced chest CT showing the right lower lobe primary tumor (approx. 3.5 cm × 2.8 cm) with ill-defined borders and multiple small interlobar pleural nodules. **(B)** After 6 cycles of pemetrexed + carboplatin + bevacizumab, CT shows marked shrinkage of the primary lesion (>60% reduction) with reappearance of a clear fat plane between the tumor and diaphragm. Pleural nodules are nearly resolved.

### 2.2 Molecular pathology and staging

Tissue-based genetic testing identified an EGFR exon 18 c.2126_2128 deletion mutation resulting in the p.E709_T710>A amino acid change (variant allele frequency 55%), co-occurring with a TP53 p.R213L mutation (39%). PD-L1 testing (22C3 pharmDx) revealed a tumor proportion score (TPS) of 55% and a combined positive score (CPS) of 55. Baseline whole-body PET-CT showed increased FDG uptake in the primary lesion (SUVmax 12.5) and pleural nodules (SUVmax 8.1), with no distant metastases. Staging was assessed as right lung adenocarcinoma cT4 (invasion of diaphragm?/pleural nodules) N0 M1a (pleural nodules), stage IVA, alongside active rheumatoid arthritis.

### 2.3 Treatment course and response

Initial Treatment and Dilemma: The first multidisciplinary team (MDT) discussion concluded that, despite the presence of an EGFR mutation, it was an uncommon type with limited prospective evidence at the time. Furthermore, although PD-L1 expression was high, the concurrent active RA posed an unknown risk for ICI therapy. After thorough discussion with the patient, initial treatment with “pemetrexed + carboplatin” chemotherapy for one cycle was chosen. The patient remained stable during this period while awaiting genetic results.

Immunotherapy Attempt and RA Flare: Upon receipt of the molecular results, and considering the high PD-L1 expression, the MDT cautiously decided to add tislelizumab (200mg) to the “pemetrexed + carboplatin” regimen for the second cycle on January 7, 2024. Within a week, the patient’s joint swelling and pain significantly worsened. Follow-up laboratory tests showed ESR increased from 28 mm/h to 61 mm/h, and CCP antibodies rose sharply from 34.5 RU/ml to 215.9 RU/ml. This was diagnosed as an ICI-triggered flare of RA activity. ICI was permanently discontinued. Management of the acute RA flare included: (1) immediate discontinuation of the offending agent; (2) methylprednisolone 1 mg/kg/day (60 mg/day IV) with rapid symptom improvement within 72 hours; (3) after symptom control, transition to oral prednisone with slow tapering (reduce by 5 mg every 2 weeks); and (4) addition of sulfasalazine (titrated to 1 g twice daily) as a long-term disease-modifying antirheumatic drug (DMARD). Hydroxychloroquine (200 mg twice daily) was also used initially. Given a history of latent tuberculosis infection (treated with prophylaxis), no biologic agent (e.g., TNF-α inhibitor) was used. By approximately 6 weeks, with prednisone tapered to 10 mg/day, RA was maintained at low disease activity (DAS28-CRP <2.6). 

Regimen Switch and Effective Induction: Starting from the third cycle on January 31, 2024, the treatment regimen was switched to “bevacizumab (500mg) + pemetrexed (0.9g) + carboplatin (600mg)” (ABCP regimen). The patient’s joint symptoms gradually improved. Treatment-related adverse events (TRAEs) during this phase were mild: grade 1 hypertension (controlled with oral irbesartan) and grade 1 proteinuria (urine protein <1 g/24h). No grade ≥3 TRAEs, bleeding, or thrombosis occurred. Restaging was performed using contrast-enhanced chest CT at baseline and after every 2 cycles (i.e., after cycles 2, 4, and 6) according to RECIST 1.1 criteria. Follow-up CT scans after 2 and 4 cycles showed continuous shrinkage of the primary lesion and pleural nodules. After six cycles, assessment confirmed a partial response (PR), with the primary lesion reduced by over 60% and pleural nodules nearly resolved (**Figure 1B**). A clear fat plane reappeared between the lesion and the diaphragm. Preoperative PET-CT showed that the primary lesion SUVmax had decreased to 3.1 (mild residual metabolic activity), and importantly, all pleural metastatic nodules achieved complete metabolic response (CMR), with no abnormal FDG uptake above background. This suggested that the chemo-antiangiogenic therapy was even more effective against the pleural metastases than against the primary lesion.

Rationale for Six Cycles of Induction: Six cycles were chosen based on the following considerations: (a) balancing efficacy and toxicity—platinum-doublet plus bevacizumab induction therapy typically achieves maximal response depth by 4–6 cycles, with additional cycles increasing cumulative toxicities without survival benefit; (b) timing of conversion surgery—the patient achieved PR after cycle 2 with sustained response through cycle 4; to maximize residual disease eradication and achieve R0 resection while avoiding chemotherapy resistance or cumulative toxicity, we proceeded with surgical evaluation after 6 cycles (the efficacy plateau).

Decision for and Execution of Conversion Surgery: A subsequent MDT reassessment concluded that the excellent systemic response had resulted in an “oligo-residual” state within the thorax, making surgical resection feasible. Bevacizumab was strictly withheld for 6 weeks preoperatively. We acknowledge that this 6-week washout period is based on pharmacokinetic reasoning (bevacizumab half-life ~20 days) and expert consensus (NCCN guidelines recommend at least 6 weeks for high-bleeding-risk surgeries), rather than prospective randomized trials in lung cancer. The FDA label suggests a longer washout (6–8 weeks). We adopted the NCCN-aligned 6-week period to balance bleeding risk (primary concern) and thrombotic risk, as the patient underwent lobectomy without planned major vascular resection. On May 16, 2024, the patient underwent an attempt at thoracoscopic right lower lobectomy and systematic mediastinal lymph node dissection. However, significant surgical challenges were encountered due to induction therapy: (1) marked frozen hilar and mediastinal lymph nodes (stations 4R, 7, 10) with dense adhesions to the pulmonary artery and superior vena cava; (2) moderate-to-severe peritumoral fibrosis and inflammatory reaction with incomplete fissures; (3) inability to safely dissect pulmonary artery branches thoracoscopically, leading to intraoperative conversion to a posterolateral thoracotomy; (4) total operative time of 285 minutes (significantly longer than routine lobectomy); and (5) approximately 400 mL of bleeding during adhesion lysis, controlled by compression and suturing. Postoperatively, the patient developed prolonged air leak (>5 days) and atrial fibrillation (controlled with medication), both likely related to extensive fibrotic dissection. Intraoperatively, visceral pleural indentation was noted. The tumor presented only as an imprint on the diaphragm without gross invasion, and the previous pleural nodules were absent.

Postoperative Pathology: Postoperative pathology confirmed moderately differentiated adenocarcinoma (3*1 cm in cross-section) with abundant foamy macrophages (treatment effect), negative bronchial margin, and metastasis in 1 of 12 lymph nodes (the positive node was a hilar node, station 10; the remaining 11 mediastinal nodes from stations 2, 4, 7, 8, and 9 were negative). The pathological stage was ypT2a (visceral pleural invasion) ypN1. According to the IASLC 2020 multidisciplinary recommendations for tumor regression grading (TRG), the TRG was Grade 1 (significant regression, defined as residual tumor cells <10%). Re-review of the original pathology slides confirmed approximately 5% residual viable tumor cells, which is fully concordant with the excellent systemic response observed on preoperative imaging (PR of primary lesion, CMR of pleural nodules) (**Figure 2**).

**Figure 2:**
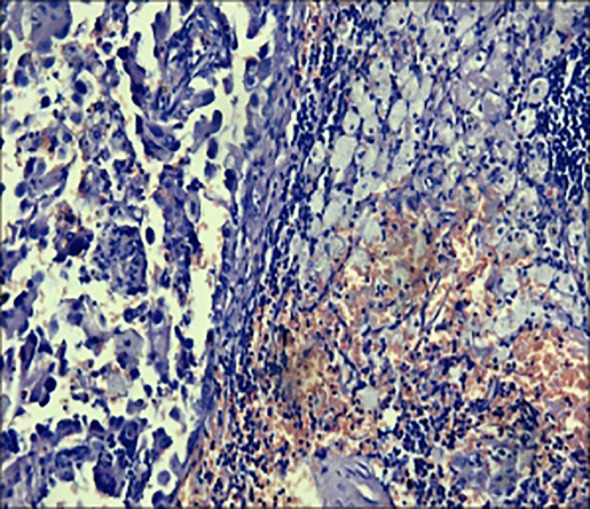
Postoperative pathology and tumor regression. Hematoxylin and eosin (H&E) staining of the resected primary tumor (original magnification ×200), highlighting a residual tumor cell nest with surrounding inflammatory infiltrate and regression changes. According to IASLC 2020 criteria, this represents TRG Grade 1 (significant regression, residual tumor cells <10%).

Postoperative Treatment and Follow-up: Maintenance therapy with “bevacizumab + pemetrexed” was resumed approximately 5 weeks post-surgery. A total of 12 cycles of maintenance therapy were administered (approximately 9 months). The decision to stop after 12 cycles was based on the patient’s no-evidence-of-disease (NED) status after R0 resection and shared decision-making to avoid overtreatment and cumulative toxicity. Regular follow-up is being conducted according to NCCN guidelines for resected NSCLC: for the first 2 years, contrast-enhanced chest CT every 3–6 months, abdominal/pelvic CT (or ultrasound) and contrast-enhanced brain MRI annually; from year 3 onward, chest CT every 6 months with annual abdominal imaging and brain MRI. Each follow-up visit also includes RA disease activity assessment (DAS28 score), treatment tolerability, and quality of life assessment. At the last follow-up (now 20 months post-operation), the patient shows no evidence of tumor recurrence. RA remains stable with low disease activity on sulfasalazine and low-dose prednisone (5 mg/day).

## 3 Discussion

This case vividly instances the “triple dilemma” faced when treating advanced NSCLC patients with active autoimmune disease and complex molecular features. It provides an exemplification for successfully navigating this challenge through a sequential, multidisciplinary strategy.

### 3.1 Therapeutic decision-making for co-existing uncommon EGFR mutation and high PD-L1 expression

This patient harbored the EGFR exon 18 E709_T710>A mutation, an uncommon variant associated with lower sensitivity to first- and second-generation TKIs. Although third-generation TKI osimertinib has shown activity against some uncommon mutations like G719X (3), prospective data specifically for this insertion-deletion mutation remained scarce at the time of treatment initiation in late 2023, and guideline recommendations were not strong. Concurrently, a PD-L1 TPS of 55% typically suggests potential benefit from ICI monotherapy. However, extensive retrospective evidence indicates that EGFR-mutant NSCLC, regardless of PD-L1 expression level, generally exhibits poor response rates (ORR <10%) and short progression-free survival to ICI monotherapy (4). This state of “double positivity but uncertain efficacy” constituted the first decision-making hurdle. Our MDT’s decision reflected the prevailing caution at the time: in the absence of overwhelming evidence favoring either targeted or immune therapy, choosing chemotherapy as the backbone was a reasonable starting point.

### 3.2 Active autoimmune disease as an “amplifier” of immune-related adverse events, not an absolute contraindication

The most direct lesson from this case is that active RA significantly increases the risk and severity of immune-related adverse events (irAEs). Just one cycle of ICI therapy precipitated a dramatic RA flare, forcing treatment cessation. This aligns with prior studies showing that patients with pre-existing AIDs have a higher incidence of any-grade and grade 3–4 irAEs compared to those without AIDs (5). However, this does not imply that all AID patients cannot receive ICIs. The critical distinction lies in disease activity. For patients with well-controlled, asymptomatic, or minimally active AID managed with low-dose steroids/conventional DMARDs, cautious use of ICIs under close monitoring may be feasible (6). This patient had active RA (high ESR, significant symptoms) at the time of lung cancer diagnosis, placing him in a high-risk category for ICI therapy. This case strongly demonstrates that initiating ICI therapy before effective control of active AID carries an unacceptably high risk. Thorough pre-treatment assessment of rheumatologic disease activity and close collaboration with rheumatology are essential safety measures.

### 3.3 Chemotherapy combined with antiangiogenic therapy: an effective conversion in a therapeutic dilemma

After the immunotherapy pathway was blocked, platinum-based chemotherapy combined with bevacizumab became the essential regimen. This strategy not only avoided triggering autoimmunity but also achieved an excellent induction response through their synergistic effects. Bevacizumab, by blocking VEGF, can normalize tumor vasculature and reduce interstitial pressure, potentially enhancing chemotherapy delivery (7). More importantly, the deep response created an opportunity for radical surgery in this initially stage IV patient. In recent years, the value of “conversion surgery” has gained increasing attention for a select subset of advanced NSCLC patients (particularly those with oligometastatic/oligo-residual disease) who exhibit exceptional responses to systemic therapy (8). The keys to success in this case were: 1) stringent MDT evaluation to ensure the patient would benefit biologically (treatment-sensitive) and anatomically (technically completely resectable); 2) strict adherence to the 6-week preoperative bevacizumab washout period, we acknowledge the evidence gap regarding this duration (discrepancy between FDA label and NCCN guidelines) and that our practice was based on pharmacokinetic reasoning and expert consensus rather than prospective randomized trials; further studies are needed to define the optimal perioperative antiangiogenic washout period in lung cancer (9); and 3) timely postoperative maintenance therapy to consolidate the response.

### 3.4 Surgical challenges after induction therapy

A critical lesson from this case concerns the significant surgical challenges posed by induction chemo-antiangiogenic therapy. The patient developed marked peritumoral fibrosis, inflammatory reactions, and frozen hilar lymph nodes, necessitating intraoperative conversion from thoracoscopy to thoracotomy, prolonged operative time (285 minutes), and increased blood loss (400 mL). Postoperative complications included prolonged air leak and atrial fibrillation. These observations align with emerging literature showing that neoadjuvant therapy, particularly immunotherapy-containing regimens, creates clinically significant inflammatory responses and fibrosis at the primary tumor site and surrounding lymph nodes, requiring longer operative times and higher conversion rates from minimally invasive to open operations. In this case, even though our regimen was chemo-antiangiogenic without immunotherapy in the neoadjuvant phase, the prior single dose of ICI and subsequent chemotherapy still induced substantial fibrosis. Therefore, for patients receiving any form of induction therapy (especially with ICI exposure), surgeons should: (1) be prepared preoperatively for possible open conversion; (2) anticipate extended operative times; (3) have strategies for managing dense adhesions; and (4) provide enhanced perioperative nutritional and rehabilitation support.

### 3.5 Neoadjuvant therapy considerations: TKI versus immunotherapy in patients with uncommon EGFR mutations and autoimmune disease

How should targeted TKI therapy and immunotherapy be chosen for neoadjuvant treatment in such complex patients? Currently, there is no established standard, and the decision must be highly individualized based on mutation type, PD-L1 expression, autoimmune disease activity, and available clinical evidence.

For patients harboring uncommon EGFR mutations (e.g., E709_T710>del, G719X, L861Q, S768I), emerging data suggest that third-generation TKIs such as osimertinib have clinical activity, although response rates are generally lower and less durable than those observed in classical EGFR mutations (exon 19 deletion or L858R) (10, 11). Neoadjuvant TKI therapy for resectable EGFR-mutant NSCLC is an area of active investigation. Several ongoing phase II trials (e.g., NCT03433469, NCT04351555, NCT04816838) are exploring the role of osimertinib as neoadjuvant or induction therapy, with preliminary results showing promising major pathological response (MPR) rates. However, these trials predominantly enroll patients with classical EGFR mutations, and data specifically for uncommon mutations remain scarce.

For patients with high PD-L1 expression, neoadjuvant immunotherapy has emerged as a powerful strategy. The CheckMate 816 trial demonstrated that neoadjuvant nivolumab plus chemotherapy significantly improved pathological complete response (pCR) and event-free survival (EFS) compared to chemotherapy alone in resectable NSCLC, regardless of PD-L1 expression (12). However, patients with active autoimmune diseases were systematically excluded from this and all other major neoadjuvant ICI trials. Real-world evidence, including our case, indicates that active RA significantly increases the risk of severe immune-related adverse events (irAEs) (5). Therefore, for patients with active autoimmune disease, neoadjuvant ICI therapy carries an unacceptably high risk and should generally be avoided unless the autoimmune disease is first brought into remission.

What about the combination of TKI and immunotherapy? Preclinical and clinical studies have shown that concurrent EGFR-TKI and ICI therapy is associated with prohibitive toxicity, particularly interstitial lung disease (incidence up to 15-30%), and is not recommended (13).

Current advancements and future directions: Several ongoing studies may provide more guidance in the coming years: Neoadjuvant TKI ± chemotherapy for uncommon EGFR mutations (e.g., UNICORN trial, NCT05425043). Novel antibody-drug conjugates (ADCs) such as patritumab deruxtecan (HER3-DXd) and trastuzumab deruxtecan (T-DXd) for rare driver-mutant NSCLC, which may offer neoadjuvant options with different safety profiles. Biomarker-driven strategies (e.g., ctDNA clearance, PD-L1 expression dynamics) to guide neoadjuvant therapy selection.

In summary, for a patient like ours with active RA and an uncommon EGFR mutation, neither neoadjuvant TKI monotherapy nor neoadjuvant ICI therapy is clearly supported by current evidence. Our chosen strategy—chemotherapy combined with antiangiogenic therapy (bevacizumab) — proved effective and safe, achieving successful conversion surgery. We eagerly await the results of ongoing prospective trials to further optimize clinical decision-making for this challenging patient population.

### 3.6 Limitations and implications of this case

This is a single retrospective case report, and extrapolation of its conclusions requires caution. Furthermore, the postoperative finding of nodal involvement (ypN1) suggests that while the primary site responded excellently, micrometastatic lesions may have been less sensitive, highlighting the potential value of surgery in eradicating resistant clones. Future prospective studies are warranted to explore how to optimize first-line strategies (e.g., potential first-line use of ADC) and more precisely select patients who would benefit from conversion surgery within this complex molecular and comorbid population.

## 4 Conclusion

This case report illustrates that initial treatment decisions for advanced NSCLC patients with concurrent uncommon EGFR mutations, high PD-L1 expression, and active autoimmune disease must be made with extreme caution. Active AID is a significant risk factor for ICI therapy. Induction therapy with chemotherapy combined with antiangiogenic agents proved effective and relatively safe in this patient, despite significant surgical challenges related to induction therapy-induced fibrosis and frozen lymph nodes that necessitated open conversion. This underscores the paramount importance of multidisciplinary collaboration and highly individualized, sequential treatment strategies in managing complex cases in the era of precision oncology for lung cancer.

## Data Availability

The datasets presented in this article are not readily available because of ethical and privacy restrictions. Requests to access the datasets should be directed to the corresponding author.

## References

[1] Passaro A Mok T Peters S Popat S . Overcoming therapy resistance in EGFR-mutant lung cancer. Nat Cancer. (2021). doi: 10.1038/s43018-021-00195-8 35122001

[2] Kennedy LC Bhatia S . Immune checkpoint inhibitors in patients with pre-existing autoimmune disease. Lancet Rheumatol. (2022). doi: 10.1080/21645515.2025.2458948 39894761 PMC11792813

[3] Cho JH . Osimertinib for patients with non–small-cell lung cancer harboring uncommon EGFR mutations. J Clin Oncol. (2022). doi: 10.1200/JCO.19.00931 31825714 PMC7098834

[4] Lee CK . Checkpoint inhibitors in metastatic EGFR-mutated non–small cell lung cancer—a meta-analysis. J Thorac Oncol. (2020). doi: 10.1016/j.jtho.2016.10.007 27765535

[5] Menzies AM . The risk of immune-related events in patients with pre-existing autoimmune disease treated with immune checkpoint inhibitors. Ann Oncol. (2021). doi: 10.1186/s41927-022-00297-5 36345032 PMC9641936

[6] Brahmer JR . Society for Immunotherapy of Cancer (SITC) clinical practice guideline on immune checkpoint inhibitor-related adverse events. J Immunother Cancer. (2021). doi: 10.1136/jitc-2021-002435 34172516 PMC8237720

[7] Liu Y Li H Wang R . Effectiveness and safety of adding bevacizumab to platinum-based chemotherapy as first-line treatment for advanced non-small-cell lung cancer: a meta-analysis. Pulmonary Med. (2021). doi: 10.3389/fmed.2021.616380 34277647 PMC8277997

[8] Yan H-J Zheng X-Y . Salvage surgery and conversion surgery for patients with nonsmall cell lung cancer: a narrative review. Int J Surg. (2025). doi: 10.1097/JS9.0000000000001921 38990285 PMC11745611

[9] Scappaticci FA . Surgical wound healing complications in metastatic colorectal cancer patients treated with bevacizumab. J Surg Oncol. (2007). doi: 10.1002/jso.20301 16118771

[10] Remon J . Osimertinib for uncommon EGFR mutations in non-small cell lung cancer. Lung Cancer. (2021). doi: 10.1016/j.lungcan.2021.03.015 33798900

[11] Passaro A . Uncommon EGFR mutations in non-small cell lung cancer. Nat Rev Clin Oncol. (2021). 37880705

[12] Forde PM . Neoadjuvant nivolumab plus chemotherapy in resectable lung cancer. New Engl J Med. (2022). doi: 10.1056/NEJMoa2202170 PMC9844511 35403841

[13] Oshima Y . EGFR-TKI plus immunotherapy for non-small cell lung cancer: a meta-analysis of safety. J Thorac Oncol. (2019). doi: 10.1016/j.jtho.2019.03.022 31002952

